# Clinical characteristics of natural recovery in trichotillomania and skin picking disorder

**DOI:** 10.3389/fpsyt.2025.1681068

**Published:** 2025-12-11

**Authors:** Megha Neelapu, Jon E. Grant

**Affiliations:** Department of Psychiatry and Behavioral Neuroscience, University of Chicago, Chicago, IL, United States

**Keywords:** trichotillomania, skin picking disorder, natural recovery, comorbidity, hair pulling

## Abstract

**Background:**

Approximately 25% of people with trichotillomania recover without receiving formal treatment. Rates of natural recovery in skin picking disorder are unknown. More importantly, variables that predict natural recovery in trichotillomania and skin picking disorder have been understudied. This study aims to examine these variables in a sample of individuals with trichotillomania and/or skin picking disorder.

**Methods:**

21 adults (76.2% trichotillomania only, 14.3% skin picking disorder only, 9.5% both; 85.7% female; mean age=32.19, SD = 10.21) who reported naturally recovering (i.e., not meeting full DSM-5 diagnostic criteria in the previous 12 months) from trichotillomania or skin picking disorder completed a virtual interview and self-report forms. The mean number of years since criteria was unmet was 5.95 (SD = 6.27). 41 participants with current trichotillomania or skin picking disorder were matched to naturally recovered participants on sex, diagnosis, and age (mean age=31.02, SD = 7.95; 2 matches for each participant except for one participant who has 1 match). They were compared on demographics, clinical characteristics, and treatment history.

**Results:**

When asked about their pulling or picking at its worst, there were no significant differences in self-reported days per week or time per day spent pulling or picking, or functional interference from pulling or picking. Participants with current trichotillomania or skin picking disorder reported greater distress from their pulling or picking, but this did not remain significant after controlling for current comorbidities. Naturally recovered participants were significantly less likely to have a current comorbid psychiatric disorder, specifically depression and ADHD. However, they were significantly more likely to have a lifetime substance use disorder and current alcohol use disorder. In total, 77.8% of participants either still pulled or picked occasionally and/or replaced it with another behavior.

**Conclusion:**

Although it may seem intuitive that those who naturally recover can do so because their disorder is less severe, these results indicate that severity was not associated with natural recovery. Given the persistence of subclinical symptoms after natural recovery, the effectiveness of natural recovery over recovery via treatment is questionable. A major limitation of this study is its small sample size and limited power. Future research should use larger sample sizes and further explore potential differences in comorbidities.

## 1 Introduction

This study aims to examine factors that may predict and lead to natural recovery in trichotillomania and skin picking disorder. Psychiatric disorders are often seen as chronic and persisting, but that may not be an accurate characterization of many disorders. Prior research has shown that many of those with alcohol use disorder, gambling disorder, depression, and obsessive-compulsive disorder (OCD) naturally recover (1–6). Similarly, Grant & Chamberlain (7) found that 24.9% of people with trichotillomania naturally recovered. Rates of natural recovery in skin picking disorder are unknown.

Why some people can naturally recover from a given disorder and others cannot remains largely unanswered for most psychiatric disorders. Grant & Chamberlain demonstrated that those who naturally recovered from trichotillomania tended to be older and had lower rates of certain comorbidities such as OCD, attention-deficit/hyperactivity disorder, panic disorder, skin picking disorder, and tic disorder. Detailed variables about their pulling behaviors, however, were not examined. Previous research on natural recovery in alcohol use disorder, gambling disorder, and OCD generally indicates that those who naturally recover tend to have a milder disorder and experience greater perceived consequences from their disorder, which may motivate recovery (8–12).

It is important to note that while natural recovery in psychiatric research is typically characterized as the complete abatement of symptoms without formal treatment, the term has no consistent definition in the literature. Defining the “natural” aspect of natural recovery lacks universal conceptual and operational definitions for any psychiatric disorder. Frank and colleagues’ 1991 article has been the basis for standardization in the field for definitions of remission and recovery, which they discuss in the context of depression (13). They define full remission as a period of time greater than the length of a depressive episode but less than a specified amount of time during which the individual is fully asymptomatic or has no more than minimal symptoms. Recovery is typically defined as long-term full remission, that is, full remission that lasts more than a specified amount of time (14).

There is sparse literature on remission and recovery for those with trichotillomania and skin picking disorder. In the little existing research, conceptual definitions on remission in trichotillomania and skin picking can broadly be characterized into two categories: 1) no longer meeting DSM-5 criteria but subclinical symptoms may persist and 2) the complete absence of symptoms (15–20). Operationally, various measures and timepoints have been used to define remission in trichotillomania and skin picking disorder, and there are no commonly agreed upon standards unlike other disorders such as depression (13, 16–19, 21). Slikboer et al. (20) have argued that recovery of trichotillomania can be conceptualized as symptom remission along with psychosocial recovery, without specifying a timeframe. However, operationally defining functional interference is challenging since it often occurs across multiple domains (social life, work, etc.), and there are no commonly agreed-upon scales to measure functional interference (22–24).

Keuthen et al. (19) previously defined current trichotillomania as a minimum trichotillomania symptom duration of 1 year with no significant remissions. Similarly, Grant & Chamberlain (7) defined recovery from trichotillomania as not meeting full criteria for the past 12 months. Thus, we may define recovery as remission that lasts for at least 1 year. In this study, we chose to define recovery as not meeting full DSM-5 criteria for trichotillomania and skin picking disorder but allowing for minimal symptoms, which is consistent with the definition used in Grant & Chamberlain’s previous study on natural recovery in trichotillomania. “Natural” was defined as a lack of effective, formal treatment for the participant’s hair pulling or skin picking. This meant that participants could receive treatment for other comorbid conditions, such as depression and anxiety. It also meant participants were allowed to have previously received treatment with no perceived benefit for their trichotillomania or skin picking disorder, but they could not have received this treatment in the past year.

It is currently unclear what factors are associated with natural recovery in trichotillomania and skin picking disorder, as variables beyond comorbidities have not been examined. Of particular interest is investigating whether or not those who naturally recover from trichotillomania and skin picking disorder have a milder disorder, similar to those with alcohol use disorder and OCD. This study aims to shed light on this topic by expanding upon the variables examined, including severity of behavior, and by using a non-recovered control group as a comparison. Clues from natural recovery can inform formal approaches for treating trichotillomania and skin picking disorder. We hypothesize that subjects who naturally recovered will have a milder disorder than those who did not (i.e., those who naturally recovered will have pulled/picked for significantly fewer days each week and spent less time pulling/picking per day than those who have been unable to naturally recover). Although some prior research on natural recovery indicates that those who naturally recover may actually experience greater perceived distress and functional interference, we hypothesize that naturally recovered participants will still report having significantly less distress and interference due to engaging in pulling/picking less frequently than those with current trichotillomania or skin picking disorder.

## 2 Methods

### 2.1 Participants

Adults aged 18 or older (*n* = 21, mean age=32.19, SD = 10.21) with lifetime but not current trichotillomania and/or skin picking disorder were recruited into the study through the TLC Foundation for Body-Focused Repetitive Behaviors website (www.brfb.org), hospital flyers, community postings, and social media posts. Each participant completed a 2-hour virtual interview and completed self-report forms via REDCap. They received $25 for their participation. Data was collected from 2021 to 2023. Natural recovery for purposes of this study was defined as not meeting full DSM-5 diagnostic criteria for trichotillomania or skin picking disorder in the previous 12 months but having met the criteria in the past, as confirmed by the TTM Diagnostic Interview – Revised for DSM-5 and Keuthen Diagnostic Interview for Skin Picking – Revised for DSM-5. Moreover, those who had received treatment for their TTM or SPD in the past year were excluded from participation. Participants may still pick or pull occasionally as long as it did not meet full criteria. None of the naturally recovered participants met any DSM-5 criteria for either disorder during the past year, except for one participant, who only met the first criterion.

Data from these naturally recovered participants were compared to participants with current DSM-5 trichotillomania and/or skin picking disorder drawn from a different study. Participants with current trichotillomania or skin picking disorder were matched to naturally recovered participants on sex, diagnosis (trichotillomania, skin picking disorder, or both disorders), and age. Two matches for each natural recovery participant were selected, except for one natural recovery participant, where only one match was found (*n* = 41, mean age=31.02, SD = 7.95). Matches were required to be within 3 years of age of the naturally recovered participants. Participants with current trichotillomania or skin picking disorder were drawn from another study conducted at the University of Chicago in 2025. These data were collected via a self-report online survey distributed in Reddit groups concerning trichotillomania, skin picking disorder, or related behaviors.

### 2.2 Assessments

#### 2.2.1 Demographics

Demographic data were collected. “Sex” was defined as biological sex assigned at birth, and “gender” was defined as current gender identity.

#### 2.2.2 Psychiatric comorbidities and treatment history

Information on lifetime comorbidities, treatment history, including lifetime medications and lifetime and current psychotherapy (and the focus of psychotherapy), was collected via self-report for all participants.

To assess current comorbidities, naturally recovered participants underwent the Mini International Neuropsychiatric Interview (MINI) English Version 7.02 for DSM-5 (25). The MINI assesses various psychiatric disorders. These include depression, mania and hypomania, panic disorder, anxiety disorders, OCD, posttraumatic stress disorder, alcohol and substance use disorders, psychotic disorders, eating disorders, antisocial personality disorder, attention-deficit/hyperactivity disorder, tic disorders, and body dysmorphic disorder.

Participants with current trichotillomania or skin picking disorder were asked to report if they had ever been diagnosed with another psychiatric disorder and could select from the following options: “I have never had a psychiatric disorder,” “depression,” “any anxiety disorder (e.g., generalized anxiety, social anxiety, panic disorder, phobias,” “PTSD,” “ADHD,” “OCD,” “bipolar disorder,” “schizophrenia,” “any personality disorder (e.g., borderline, narcissistic, avoidant),” “eating disorder,” “alcohol use disorder,” “substance use disorder,” and “other.” Subjects were then asked if they were currently experiencing symptoms of each disorder that was selected to assess current comorbidities.

#### 2.2.3 Trichotillomania and skin picking disorder clinical assessments

For naturally recovered participants, the Trichotillomania Diagnostic Interview – Revised for DSM-5 or the Keuthen Diagnostic Interview for Skin Picking – Revised for DSM-5 were used to verify that they met lifetime but not current diagnostic criteria. Naturally recovered participants also completed a self-report questionnaire about the history of their pulling/picking, including between what ages disorder criteria were met, past severity of pulling/picking (i.e. days spent pulling/picking each week and time each day spent pulling/picking), reasons for not receiving treatment, and whether they continued to engage in any hair-pulling or skin picking (albeit at a lower rate) after they no longer met full diagnostic criteria.

All participants also reported on clinical variables related to pulling and picking, including age of onset. If a range was given for time per day spent pulling or picking, the minimum value of that range was used in analyses. Distress and functional interference from trichotillomania and skin picking disorder were assessed via two self-report items: “When your pulling or picking was at its worst, how much did the behavior bother you?” and “When your pulling or picking was at its worst, how much did the behavior interfere with your life?” Additionally, participants with current trichotillomania or skin picking disorder reported on current (over the past week) clinical characteristics of their pulling and picking. These consisted of days spent pulling or picking in the past week, time per day spent pulling or picking, distress from pulling or picking, and functional interference from pulling or picking.

### 2.3 Data analysis

Descriptive statistics were derived for naturally recovered participants on details about their past trichotillomania or skin picking disorder, and on current pulling and picking details for participants with current trichotillomania and skin picking disorder.

Naturally recovered participants were compared to those with current trichotillomania or skin picking disorder on demographic variables, pulling and picking details, psychiatric comorbidities, and treatment history using independent samples t-tests, analyses of variance and covariance (ANCOVAs controlled for current comorbidities), or chi-square tests as applicable. The appropriate effect size was used for each test (Cohen’s D for t-tests, partial eta squared for ANOVAs and ANCOVAs, and Phi for chi-square tests).

To compare distress and interference from pulling and picking, the scores of naturally recovered participants were divided by two and rounded down to the nearest whole number to convert the 0–10 scale to a 0–5 scale to be consistent with the items that were used for non-recovered participants (e.g., a score of 9 became a score of 4).

All analyses and effect sizes were conducted with IBM SPSS Statistics Version 31.0.0.0. Statistical significance was defined as *p* < 0.05.

## 3 Results

### 3.1 Demographics

Twenty-one adults (85.7% female; mean age=32.11, SD = 10.21) who naturally recovered from trichotillomania or skin picking disorder completed a virtual interview and self-report forms and were matched to 41 participants with current trichotillomania or skin picking disorder (85.4% female; mean age=31.02, SD = 7.95).

The sample of participants with current trichotillomania or skin picking disorder had a significantly greater proportion of non-white participants than the naturally recovered sample, X^2^(1, *n* = 59) = 5.110, p=.043, with a small effect size (φ=.294). Naturally recovered participants did not significantly differ from participants with current trichotillomania or skin picking disorder on any other demographic variable (**Table 1**).

**Table 1:** Demographics for a sample of 62 people with trichotillomania or skin picking disorder, stratified by group.

		Group				
		Naturally recovered *M (SD)* or %	Current trichotillomania or skin picking disorder *M (SD)* or %	T-statistic or Chi-square	*P*	Cohen’s D or Phi
Age in years (*n* = 62)		32.19 (10.21)	31.02 (7.95)	.496	.622	.133
Sex (*n* = 62)	Female	85.7%	85.4%		1.000	.005
	Male	14.3%	14.6%			
Gender (*n* = 62)	Woman	90.5%	85.4%		.705	.072
	Man	9.5%	14.6%			
Ethnicity (*n* = 48)	Non-Hispanic/Latino	100%	90.6%		.541	.183
	Hispanic/Latino	0%	9.4%			
Race, simplified (*n* = 59)*	White	95.0%	69.2%	5.110	.043	.294
	Non-white	5.0%	30.8%			
Sexual orientation (*n* = 59)	Straight	60.0%	66.7%	.426	.935	.085
	Gay or lesbian	10.0%	7.7%			
	Bisexual or pansexual	25.0%	23.1%			
	Other	5.0%	2.6%			
Educational attainment (*n* = 57)	High school diploma or GED	10.5%	0%	6.938	.074	.349
	Some college or associate’s degree	26.3%	13.2%			
	Bachelor’s degree	26.3%	50.0%			
	Master’s degree or higher	36.8%	36.8%			
Employment status (*n* = 59)	Full-time	55.0%	66.7%	3.474	.482	.243
	Part-time	15.0%	7.7%			
	Student	20.0%	17.9%			
	Unemployed	10.0%	2.6%			
	Other	0%	5.1%			
Marital status (*n* = 62)	Single	52.4%	56.1%	3.326	.505	.232
	Married	33.3%	24.4%			
	Divorced	4.8%	7.3%			
	Engaged	4.8%	12.2%			
	Widowed	4.8%	0%			

** indicates a statistically significant difference in variable distribution between groups (*p* <.001); * indicates a statistically significant difference in variable distribution between groups (*p* <.05). Fisher’s Exact Tests were used instead of Chi-Square Tests when more than 20% of cells had expected cell counts of less than 5.

### 3.2 Trichotillomania and skin picking disorder clinical variables

For naturally recovered participants, 76.2% had trichotillomania only, 14.3% had skin picking disorder only, and 9.5% had both disorders. For those who naturally recovered, the mean age at which DSM-5 criteria were met was 13.81 (*SD* = 5.65) and unmet was 26.60 (*SD* = 8.78). The mean number of years of meeting diagnostic criteria was 13.10 (*SD* = 8.77) and the mean number of years since criteria was unmet was 5.95 (*SD* = 6.27). 61.1% of participants pulled or picked occasionally after they no longer met full criteria for trichotillomania or skin picking disorder. 55.6% of participants answered “yes” to the question, “If you have stopped picking or pulling entirely, has anything taken its place? (e.g., nail biting)” Answers included popping pimples, binge eating, applying pressure to fingernails, nail biting, mild lip picking (for a participant previously diagnosed with trichotillomania), self-injury, rocking, knee bouncing, and increased phone use. In total, 77.8% of participants either still pulled or picked occasionally and/or replaced it with another behavior. The most common response to the question “Why did you not receive treatment for the condition at the time (check all that apply)?” was participants reporting that they received unsuccessful treatment (26.3%), followed by the answer “I sought treatment but no treatment was available to me” (15.8%) (**Table 2**).

**Table 2:** Descriptive statistics for 21 naturally recovered participants.

		M (SD) or %
Age criteria met (*n* = 21)		13.81 (5.65)
Age criteria unmet (*n* = 20)		26.60 (8.78)
Years criteria met (*n* = 20)		13.10 (8.77)
Why did you not receive treatment for the condition at the time (tick any that applied)? (*n* = 19)	I sought treatment but no treatment was available to me.	15.8%
	I didn’t realize trich or picking was a disorder that had treatments.	10.5%
	I felt that the symptoms would get better on their own.	5.3%
	I avoided seeking treatments because I was embarrassed/ashamed of my problem.	10.5%
	Received treatment, didn’t help (write-in)	26.3%
	Other***	31.6%
Starting after the time you no longer met criteria, have you pulled/picked at all? (*n* = 18)		61.1%
If you have stopped picking/pulling entirely, has anything taken its place? (e.g. nail biting) (*n* = 18)		55.6%

***Other reasons: Treated self on own, had treatment primarily for other issues, moved around a lot, never sought treatment (Jehovah’s witness).

Participants with current trichotillomania and skin picking disorder also reported on pulling or picking behaviors over the past week, including days spent pulling or picking, time spent per day, and distress and functional interference (**Table 3**).

**Table 3:** Descriptive statistics on current pulling and picking for 41 participants with current trichotillomania or skin picking disorder.

	*M (SD)* or %
Days pulling in the past week (*n* = 35)	5.83 (1.40)
Typical time per day pulling in the past week, in minutes (*n* = 35)	95.69 (131.54)
Distress from pulling in the past week (0-5) (*n* = 35)	3.97 (1.15)
Functional interference from pulling in the past week (0-5) (*n* = 35)	2.83 (1.44)
Pulling currently at worst? (*n* = 35)	40.0%
Days picking in the past week (*n* = 10)	6.20 (1.32)
Typical time per day picking in the past week, in minutes (*n* = 10)	92.50 (90.87)
Distress from picking in the past week (0-5) (*n* = 10)	4.00 (1.05)
Functional interference from picking in the past week (0-5) (*n* = 10)	3.10 (1.52)
Picking currently at worst? (*n* = 10)	50.0%

Naturally recovered participants’ self-reported score on how much their pulling or picking bothered them when it was at its worst (*M* = 4.17) was significantly lower than those with current trichotillomania or skin picking disorder (*M* = 4.63), with a medium effect size (η^2p^=.076). However, this result did not remain significant after controlling for current comorbidities, including current depression. There was no significant difference in days per week or time per day spent pulling or picking, functional interference from pulling or picking, or age of onset of pulling or picking (**Table 4**).

**Table 4:** Clinical variables for 62 adults with trichotillomania and/or skin picking disorder stratified by group.

		Group				
		Naturally recovered *M (SD)* or %	Current trichotillomania or skin picking disorder *M (SD)* or %	T-statistic, F-statistic or Chi-square	*P*	Cohen’s D, partial eta squared or Phi
Diagnosis (*n* = 62)	TTM only	76.2%	75.6%	.003	.999	.006
	SPD only	14.3%	14.6%			
	TTM and SPD	9.5%	9.8%			
Age at first pulling or picking (*n* = 56)		14.11 (6.52)	11.73 (5.49)	1.438	.156	.406
Age at first pulling (*n* = 47)		14.63 (6.80)	12.71 (5.21)	1.075	.288	.331
Age at first picking (*n* = 12)		11.50 (3.87)	9.25 (6.14)	.662	.523	.405
Pull or pick every day (*n* = 56)		89.5%	83.8%		.703	-.077
Pull every day (*n* = 47)		87.5%	80.6%		.697	-.086
Pick every day (*n* = 12)		75.0%	87.5%		1.000	.158
Time per day pulling or picking, in minutes (*n* = 53)		123.61 (111.33)	143.63 (138.15)	-.532	.597	-.154
Time per day pulling, in minutes (*n* = 44)		118.00 (118.60)	132.48 (117.63)	-.359	.721	-.114
Time per day picking, in minutes (*n* = 12)		112.50 (92.87)	161.25 (197.17)	-.461	.655	-.282
Distress from pulling or picking (0-5)	Uncontrolled (*n* = 53)*	4.17 (1.04)	4.63 (.60)	4.217	.045	.076
	Controlled for any current comorbidity (*n* = 53)	4.28	4.57	1.518	.224	.029
	Controlled for current depression (*n* = 53)	4.24	4.59	2.132	.151	.041
Distress from pulling (0-5) (*n* = 38)		4.31 (1.18)	4.68 (.48)	-1.090	.294	-.474
Distress from picking (0-5) (*n* = 9)		4.00 (.00)	4.17 (.98)	-.415	.695	-.201
Functional interference from pulling or picking (0-5) (*n* = 53)		3.17 (1.47)	3.60 (1.38)	-1.062	.293	-.308
Functional interference from pulling (0-5) (*n* = 38)		3.23 (1.54)	3.52 (1.39)	-.588	.560	-.201
Functional interference from picking (0-5) (*n* = 9)		3.00 (1.73)	3.17 (1.47)	-.152	.883	-.107

* indicates a statistically significant difference in variable distribution between groups (*p* <.05). Fisher’s Exact Tests were used instead of Chi-Square Tests when more than 20% of cells had expected cell counts of less than 5. If subject met criteria for both TTM and SPD and gave two different responses for each then the higher value was used.

### 3.3 Comorbidities and treatment history

Naturally recovered participants were significantly less likely to have any current psychiatric comorbidity, X^2^(1, *n* = 62) = 7.201, p=.007, with a medium effect size (φ=.341) (see **Table 5**). Specifically, they were significantly less likely to have current depression, X^2^(1, *n* = 62) = 7.345, p=.007, with a medium effect size (φ=.344), and current ADHD, X^2^(1, *n* = 60) = 10.838, p=<.001, with a medium effect size (φ=.425). Non-white participants were significantly more likely to have current depression, so the chi-square test was repeated among white participants only and non-white participants only. While neither result was significant, rates of current depression remained higher for participants with current trichotillomania and skin picking disorder for both white and non-white participants. Naturally recovered participants were also significantly more likely to have a lifetime substance use disorder, X^2^(1, *n* = 62) = 5.167, p=.041, with a small effect size (φ=-.289), and current alcohol use disorder, X^2^(1, *n* = 62) = 6.155, p=.035, with a medium effect size (φ=-.315).

**Table 5:** Psychiatric comorbidities and treatment history in 62 adults with trichotillomania and/or skin picking disorder, stratified by group.

		Group				
		Naturally recovered %	Current trichotillomania or skin picking disorder %	Chi-square	*P*	Phi
Any lifetime comorbidity (*n* = 62)		90.5%	80.5%		.472	-.129
Lifetime depression (*n* = 62)		81.0%	65.9%	1.537	.215	-.157
Lifetime anxiety disorder (*n* = 62)		71.4%	63.4%	.398	.528	-.080
Lifetime PTSD (*n* = 62)		14.3%	17.1%		1.000	.036
Lifetime ADHD (*n* = 62)**		4.8%	46.3%	10.987	<.001	.421
Lifetime OCD (*n* = 62)		9.5%	19.5%		.472	.129
Lifetime eating disorder (*n* = 62)		14.3%	9.8%		.680	-.068
Lifetime AUD (*n* = 62)		19.0%	4.9%		.167	-.227
Lifetime SUD (*n* = 62)*		19.0%	2.4%		.041	-.289
Any current comorbidity (*n* = 62)*		38.1%	73.2%	7.201	.007	.341
Current depression (*n* = 62)*	All (*n* = 62)*	4.8%	36.6%	7.345	.007	.344
	White (*n* = 46)	5.3%	29.6%	4.207	.061	.302
	Non-white (*n* = 13)	0%	58.3%	1.264	.462	.312
Current anxiety disorder (*n* = 62)		28.6%	53.7%	3.529	.060	.239
Current PTSD (*n* = 62)		0%	4.9%		.545	.131
Current ADHD (*n* = 62)**		5.0%	47.5%	10.838	<.001	.425
Current OCD (*n* = 62)		0%	17.1%		.084	.255
Current eating disorder (*n* = 60)		5.0%	7.5%		1.000	.047
Current AUD (*n* = 62)*		14.3%	0%		.035	-.315
Current SUD (*n* = 62)		9.5%	0%		.111	-.255
Any lifetime treatment (*n* = 62)*		52.4%	85.4%	7.891	.005	.357
Lifetime treatment for TTM or SPD (*n* = 57)		26.3%	52.6%	3.563	.059	.250
Lifetime psychiatric medication use (*n* = 62)		71.4%	70.7%	.003	.954	-.007
Lifetime psychotherapy (*n* = 62)		52.4%	73.2%	2.680	.102	.208
Current psychotherapy (*n* = 62)*		14.3%	39.0%	3.999	.046	.254

* indicates a statistically significant difference in variable distribution between groups (*p* <.05). Fisher’s Exact Tests were used instead of Chi-Square Tests when more than 20% of cells had expected cell counts of less than 5.

Participants with current trichotillomania and skin picking disorder were significantly more likely to have received lifetime treatment for any psychiatric symptoms (not just for trichotillomania or skin picking disorder), X^2^(1, *n* = 62) = 7.891, p=.005, with a medium effect size (φ=.357), as well as to be currently receiving psychotherapy for any psychiatric symptoms, X^2^(1, *n* = 62) = 3.999, p=.046, with a small effect size (φ=.254).

## 4 Discussion

The results from this study suggest that those with trichotillomania and skin picking disorder who naturally recover do not have a less severe disorder than those who continue to struggle with their pulling or picking, contrary to our hypothesis and much of the literature on natural recovery in other psychiatric disorders. Thus, this study indicates that severity may not be the best measure in predicting outcomes for those with trichotillomania and skin picking disorder. While this study proposes other factors that may be better predictors, such as comorbidities, further research is needed. It is important to note that these findings should be viewed as preliminary given that this study relied on retrospective recall of pulling and picking behaviors.

In this study, there was no significant difference in the days per week or time per day spent pulling or picking, or from functional interference from pulling or picking, when the behavior was at its worst in the participants’ lifetime. Those with current trichotillomania and skin picking disorder did report their pulling or picking as more bothersome than those who naturally recovered when it was at its worst. However, these results did not remain significant after controlling for current comorbidities, including current depression. There are multiple potential interpretations of this result. One may be that depressive symptoms worsen distress from pulling and picking. Grant and colleagues (26) found that comorbid depression may exacerbate multiple variables related to hair pulling, including severity, ability to resist urges, and functional interference. A different interpretation is that participants with current depressive symptoms are overestimating their recall of their distress. There is a great deal of evidence that demonstrates that current depressive symptoms lead to exaggerated retrospective reports of negative affect (27–29). Given that no significant differences were observed in other clinical characteristics related to hair pulling and skin picking, this explanation may be stronger than the prior one.

Naturally recovered participants indicated significantly lower rates of current comorbidities. Findings regarding comorbidities in this study should be interpreted with caution due to differences in how they were reported, as current comorbidities were assessed with the MINI by a clinician for the naturally recovered participants, while comorbid diagnoses were self-reported by participants with current trichotillomania and skin picking disorder. The differences in rates of alcohol use disorder and substance use disorders should further be evaluated carefully due to low sample size. Nevertheless, the finding of lower rates of comorbidities in the naturally recovered participants is consistent with most research on natural recovery for other disorders and prior research on natural recovery in trichotillomania (7, 11, 30). Comorbidities can make natural recovery as well as recovery via treatment more challenging, as different disorders may exacerbate one another.

Specifically, naturally recovered participants had significantly lower rates of current depression and ADHD. Depressive symptoms have previously been found to be associated with worse pulling and picking symptoms (26, 31, 32). Those with depression may find giving up pulling and picking more difficult as they are experiencing more negative emotions and distress, and they are using pulling and picking as a maladaptive coping mechanism (33, 34). Current research on potential links between ADHD and trichotillomania and skin picking disorder is minimal and the results are mixed (35–38). Ultimately, additional research is needed to determine the nature of ADHD in trichotillomania and skin picking disorder and its potential relationship with natural recovery.

The effectiveness of natural recovery over recovery via treatment is questionable. Among those who naturally recovered, most continued to report pulling or picking at non-diagnostic levels and/or replaced their pulling or picking with another possibly unhealthy or harmful behavior. These statistics may indicate that those with trichotillomania and skin picking disorder are choosing natural recovery over formal treatment because one can attempt to control, moderate, or reduce one’s use, rather than quit completely. Furthermore, these results are in line with studies done on problematic drinking, which show that untreated remitters are more likely to be non-abstinent and continue drinking in moderation than treated remitters (12, 39). Over a third of those who naturally recovered reported seeking treatment but not finding any or receiving ineffective treatment, which signals that lack of access to effective treatments may also be an aspect in natural recovery. Formal treatment may prevent the occasional pulling and picking and help participants avoid replacing their pulling and picking with a different unhealthy behavior. One study in trichotillomania demonstrated that immediately after formal treatment, abstinence from pulling predicted a greater likelihood of maintenance at a 3-month follow-up (17). A direction for future research could be comparing those who remit and recover naturally to those who do so via treatment to assess the effectiveness of each approach.

Moreover, these results once again bring into question how we should define recovery. Are individuals who continue to minimally pull or pick or have replaced it with another destructive habit truly recovered? Another area for future research may be comparing psychosocial functioning between those who recovered who have minimal symptoms and those who are completely abstinent. Moreover, if we choose to set stricter definitions for remission and recovery, how does this change our treatment approach? Habit reversal training, which is the first-line treatment for trichotillomania and skin picking disorder, focuses on forming awareness of the habit, avoiding triggers, and responding to urges with a competing response (e.g., clenching one’s fists to prevent pulling or picking) (40–42). However, while habit reversal training helps with symptom reduction, it may not address underlying factors that lead to pulling and picking. Leaving these unaddressed may cause continued subclinical symptoms or replacing the behavior with another harmful habit. Therefore, pairing habit reversal training with cognitive behavioral therapy or other evidence-based psychotherapies can help with emotion dysregulation and dysfunctional beliefs that underpin pulling and picking. Moreover, psychiatric medications may help manage neurological factors of pulling and picking behaviors (40, 42, 43).

This study has several limitations. Primarily, the reporting of hair pulling and skin picking clinical characteristics relied on retrospective recall. Another is that our definition of the “natural” aspect of natural recovery as the absence of effective, formal treatment is a subjective one. For future research, longitudinal studies that follow those with trichotillomania and skin picking disorder can more accurately measure pulling and picking behaviors and related psychiatric symptoms as well as determine the effectiveness or lack thereof of treatments. They can also answer many of the questions raised by this study, including whether recovery via treatment or natural recovery leads to better outcomes for those with trichotillomania and skin picking disorder. Another major limitation was that this was a very small study, the purpose of which was to investigate whether there were any variables linked to natural recovery worth exploring further. Partly due to this limited size, the sample was majority female, non-Hispanic white, and highly educated. Future studies should use larger, more diverse samples when exploring significant variables discussed in this study. It may also be worth separating those with trichotillomania from skin picking disorder rather than grouping them in analysis to capture potential differences in natural recovery between these two groups.

## 5 Conclusions

Individuals who naturally recover from trichotillomania and skin picking disorder do not appear to have a milder disorder than those who do not naturally recover. Those who naturally recover may have fewer overall comorbidities, but more research is needed on the potential effects of specific comorbidities. Roughly three-quarters of naturally recovered participants still replaced their pulling or picking with another unhealthy behavior or still pulled or picked to a lesser extent, indicating that recovery via treatment may still be more effective. While natural recovery as defined in this study allowed for continued subclinical symptoms, it is worth considering stricter definitions in future research. Giving up pulling and picking is often quite difficult for people to imagine. Moreover, formal treatment for trichotillomania and skin picking disorder is often inaccessible. There is currently no FDA-approved medication treatment for either disorder, and finding trained therapists in behavioral therapy for these disorders is difficult (44, 45). Formal treatment may be viewed as more appealing than self-change when there is an effective medication available. Ultimately, this study highlights the importance of making treatment more accessible and gives insight into factors associated with recovery that can inform treatment practices.

## Data Availability

The original contributions presented in the study are included in the article/supplementary material, further inquiries can be directed to the corresponding author/s.
